# Acquired Pediatric Right Diaphragmatic Hernia Following Automatic Implantable Cardioverter-defibrillator Placement

**DOI:** 10.5811/cpcem.2019.9.43992

**Published:** 2019-10-21

**Authors:** Adria Ottoboni, Larissa Morsky, Laura C. Castro, Mark Rhoades, Daniel Quesada, Phillip Aguìñiga-Navarrete

**Affiliations:** * Kern Medical Center, Department of Emergency Medicine, Bakersfield, California; † LA+USC Medical Center, Department of Emergency Medicine, Los Angeles, California

## Abstract

Diaphragmatic hernias are an uncommon occurrence in the pediatric population; however, they can cause significant morbidity and mortality if the diagnosis is missed or delayed. This case discusses the radiographic and clinical exam findings of a one-year-old patient with this pathology.

## CASE PRESENTATION

A one-year-old male with a past medical history of prolonged QT syndrome and past surgical history of a sternotomy for automatic implantable cardioverter-defibrillator (AICD) and pacemaker placements presented to the emergency department (ED) in moderate respiratory distress with tachypnea, nasal flaring, and subcostal retractions. The father reported the patient had been irritable, coughing, vomiting, and diaphoretic over the prior day.

**Image 1:**
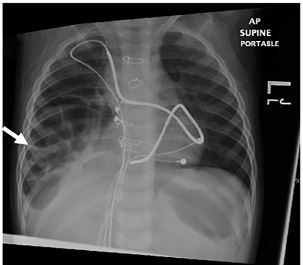
Anteroposterior chest radiograph (CXR) performed on a child who presented to the emergency department in respiratory distress; CXR demonstrated significant elevation of the right hemidiaphragm with colonic interposition and right lung base infiltrate (arrow).

**Image 2:**
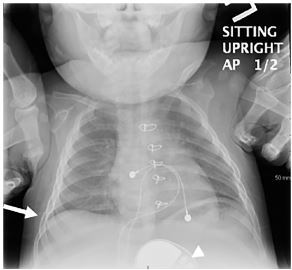
Anteroposterior chest radiograph demonstrates normal right-sided costophrenic angle and prior stemotomy without any elevation of the diaphragm (arrow). Also visualized is the pulse generator placed in the abdomen (arrowhead).

Chest radiograph (CXR) demonstrated infiltrate versus atelectasis in the right lung base with a large right-sided diaphragmatic hernia demonstrated by infiltration of the large bowel into the right hemithorax with apparent bowel gas in the chest cavity (Image 1). CXR from six months prior showed no evidence of diaphragmatic hernia (Image 2). Prior to transfer to higher level of care, the patient received fluid resuscitation, supplemental oxygen, nebulized albuterol, and antibiotics for presumed pneumonia. His tachypnea and hypoxia subsequently improved. During operative intervention, findings included herniation of the small bowel, colon, and omentum through the right-sided diaphragmatic defect in the anterior subxiphoid region, with the liver displaced posteriorly. The pacemaker coil leads were wrapped within this hernia.

## DISCUSSION

**Image 3:**
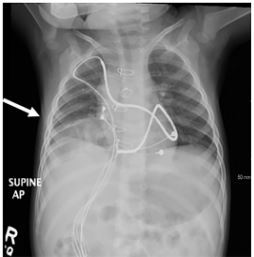
Anteroposterior chest radiograph one month prior to image 1, demonstrating right colonic interposition with probable associated atelectasis (arrow).

In this case, the right-sided diaphragmatic hernia was likely a sequelae of the initial surgical placement of the AICD. As seen on CXR (Image 3) from approximately one month prior to presentation in the ED, the diaphragmatic hernia slowly increased in size and demonstrated colonic interposition. The patient presented with nausea and vomiting but was asymptomatic from a respiratory standpoint; therefore, no intervention was pursued as he had close follow-up with an outside facility. The hernia eventually progressed, causing respiratory distress and ventilation-perfusion mismatch. This manifested as an increased respiratory rate, evidence of auto-peep, and feeding difficulties.

While uncommon in children, patients with diaphragmatic hernias may present with respiratory distress, unspecified thoracoabdominal pain, or symptoms of gastrointestinal obstruction such as nausea and vomiting. Herniation of abdominal contents into the chest cavity may also be identified by auscultation of bowel sounds in the chest.^1^,^2^ The patient should be radiographically examined quickly upon presentation to avoid the morbidity and mortality associated with delayed diagnosis and management.^1^,^2^

**CPC-EM Capsule**

What do we already know about this clinical entity?

*Right-sided diaphragmatic hernias are difficult to diagnose in children, and diagnostic delay contributes to morbidity and mortality*.

What is the major impact of the image(s)?

*Diagnosis of right-sided diaphragmatic hernia, a likely sequela of automatic implantable cardioverter-defibrillator (AICD) placement, was made via chest radiograph*.

How might this improve emergency medicine practice?

*Children presenting with an AICD presenting respiratory distress should warrant suspicion of right diaphragmatic hernia and be radiographically examined to avoid mortality*.
